# AI-Integrated Multi-Target Validation of *Coreopsis tinctoria* Polyphenols as a Functional Food Ingredient Against Diabetic Nephropathy

**DOI:** 10.3390/foods15132257

**Published:** 2026-06-23

**Authors:** Dilinare Abdurehman, Xueying Lu, Yindengzhi Guoruoluo, Geyu Liu, Jun Li, Tao Wu, Xuelei Xin, Haji Akber Aisa

**Affiliations:** 1 State Key Laboratory Basis of Xinjiang Indigenous Medicinal Plants Resource Utilization and Key Laboratory of Plants Resources and Chemistry of Arid Zone, Xinjiang Technical Institute of Physics and Chemistry, Chinese Academy of Sciences, Urumqi 830011, China; dilinare@ms.xjb.ac.cn (D.A.); xueyinglu@ms.xjb.ac.cn (X.L.); yindz@ms.xjb.ac.cn (Y.G.); liugy@ms.xjb.ac.cn (G.L.); lijun702@ms.xjb.ac.cn (J.L.); wutao@ms.xjb.ac.cn (T.W.); xinxl@ms.xjb.ac.cn (X.X.); 2 University of Chinese Academy of Sciences, Beijing 100049, China; 3 Institute of Traditional Chinese Medicine, Xinjiang Medical University, Urumqi 830054, China

**Keywords:** *Coreopsis tinctoria* Nutt., diabetic nephropathy, machine learning, polyphenols, inflammation, renal fibrosis

## Abstract

Diabetic nephropathy (DN) is a severe diabetic complication with substantial clinical burden. The complex pathogenesis of DN has hindered the development of targeted therapies, creating an urgent need to develop novel strategies that directly address its underlying inflammatory and fibrotic mechanisms. *Coreopsis tinctoria* (CE) is an edible plant rich in polyphenols, but its mechanism against DN remains understood. An integrated framework combining network pharmacology and machine learning was developed to prioritize active polyphenols and their targets. A multi-layer perceptron classifier, trained on 3.16 million compound–target pairs from Binding DB, predicted interactions between 36 CE polyphenols and 12,030 DN-associated genes. The top 100 targets were subjected to KEGG enrichment analysis, and the identified pathways were validated in a high-fat diet/STZ-induced DN rat model. The MLP model achieved superior performance (AUC-ROC = 0.9219, AP = 0.9592). Five lead polyphenols (flavonoids/chalcones) showed high predicted activity. KEGG analysis revealed enrichment in PI3K-Akt, calcium signaling, metabolic pathways, and cellular senescence. In vivo, CE treatment (150–600 mg/kg/day) dose-dependently improved glucose/lipid metabolism and renal function, and ameliorated histopathological damage, including glomerular hypertrophy, fibrosis, and mesangial expansion. Mechanistically, CE suppressed NF*κ*B/TGF*β*/Smad signaling, restored PPAR*γ* and Nrf2/HO-1/FoxO1 antioxidant defenses, and inhibited apoptosis via Bcl-2/Bax regulation. CE exerts multi-target renoprotective effects through coordinated modulation of metabolic, inflammatory, fibrotic, and antioxidant pathways, supporting its potential as a functional food ingredient for DN management.

## 1. Introduction

Diabetic nephropathy (DN) is a leading cause of end-stage renal disease worldwide, affecting approximately 30–40% of patients with diabetes [1]. Despite advances in glycemic control and blood pressure management, the residual risk of DN progression remains substantial, underscoring the urgent need for novel preventive and therapeutic strategies. The pathogenesis of DN is multifactorial, involving hyperglycemia-induced metabolic dysregulation, oxidative stress, chronic inflammation, and accumulation of advanced glycation end products (AGEs) [2]. These interrelated pathological processes converge on key signaling pathways that promote glomerular mesangial expansion, basement membrane thickening, extracellular matrix deposition, and ultimately, renal fibrosis [3]. Among these, the TGF*β*/Smad signaling axis plays a central role in driving fibrogenesis, while NF*κ*B activation underpins the inflammatory cascade that exacerbates renal injury [4]. Given the complexity of DN pathology, therapeutic strategies that simultaneously target multiple pathogenic pathways are likely to be more effective than single-target approaches.

Nutritional interventions have gained increasing attention as complementary strategies for DN management, owing to their multi-target nature and favorable safety profiles [5]. Peroxisome proliferator-activated receptor gamma (PPAR*γ*) is a key nuclear receptor that integrates metabolic, anti-inflammatory, and anti-fibrotic signals. PPAR*γ* activation not only improves insulin sensitivity and lipid metabolism but also suppresses NF*κ*B-mediated inflammatory gene transcription, highlighting its potential as a therapeutic node in DN [6,7]. Similarly, the Nrf2/HO-1 antioxidant pathway counteracts oxidative stress, while FoxO transcription factors regulate cellular stress resistance and metabolic homeostasis. Thus, natural products capable of modulating these pathways hold promise as functional food ingredients for DN prevention.

*Coreopsis tinctoria* Nutt., a perennial herb indigenous to southern Xinjiang, China, has been traditionally used as both a medicinal tea and dietary component [8]. It is rich in flavonoids and phenolic compounds with well-documented antioxidant, anti-inflammatory, anti-diabetic, and lipid-regulating properties [9,10,11]. Recent studies have shown that *C. tinctoria* extracts activate Nrf2 and PPAR*γ*, contributing to protection against metabolic and inflammatory disorders [12]. Our previous phytochemical profiling identified 74 compounds, predominantly flavonoids and phenolics, including isookanin-7-*O-β*-D-glucoside and marein with bioactivity relevant to glucose and lipid metabolism [13]. Moreover, *C. tinctoria* has been reported to improve insulin resistance and attenuate renal inflammation and fibrosis in in vitro and in vivo models [4]. However, the precise mechanisms by which *C. tinctoria* polyphenols exert renoprotective effects—particularly which compounds and molecular pathways are primarily responsible—remain incompletely understood.

Traditional experimental approaches, which often focus on individual compounds or single pathways, are insufficient to capture the multi-component, multi-target nature of botanical extracts. In recent years, network pharmacology has emerged as a powerful tool for mapping system-level bioactivity networks and identifying critical target pathways [14]. However, conventional network pharmacology methods often rely on simple molecular docking or literature-derived target databases, which may lack predictive power or introduce bias toward well-studied targets. To address these limitations, supervised machine learning (ML) algorithms trained on large-scale experimentally validated compound–target binding data can learn complex non-linear relationships between molecular features and binding outcomes, thereby enabling the prediction of novel compound–target interactions with greater accuracy [15]. This approach is particularly valuable for natural product research, where chemical diversity is high and experimental target identification remains a bottleneck. ML-based prioritization also reduces the dimensionality of complex herbal systems, allowing researchers to focus experimental resources on the most promising compound–target pairs.

Based on these considerations, the present study aimed to (1) employ an AI-integrated network pharmacology framework to prioritize active polyphenols from *C. tinctoria* and predict their targets associated with DN; (2) validate the identified pathways in a high-fat diet/STZ-induced DN rat model; and (3) elucidate the underlying molecular mechanisms, with particular focus on metabolic regulation, inflammation, fibrosis, and oxidative stress. This study provides a scientific foundation for developing *C. tinctoria* as a functional food ingredient for the prevention and management of DN.

## 2. Materials and Methods

### 2.1. Chemicals and Materials

Streptozotocin (STZ, No. S0130) was purchased from Sigma-Aldrich. Serum biochemical detection kits were obtained from Mindray Ltd. (Shenzhen, China). ELISA kits for cytokines TNF-*α* (No. YX-E21174), IL-1*β* (No. YX-E28967), and IL-6 (No. YX-E21185) were from Sinobestbio Biotechnology Ltd. (Shanghai, China). Rat AGEs ELISA kit (No. CSB-E09413r) was supplied by Cusabio Biotechnology, Inc. (Wuhan, China). Rat insulin ELISA kit (No. RX302147R) was from Raysun Biotechnology Ltd. (Guangzhou, China). 2× QuantiNova SYBR Green PCR Master Mix (No. 208054) was purchased from QIAGEN (Hilden, North Rhine-Westphalia, Germany). The cDNA synthesis kit and TRIzol reagent were from TIANGEN (Beijing, China). Primary antibodies used in this study are listed in Table S1.

### 2.2. Sample Preparation

CE (*C. tinctoria* extract) was prepared as previously described [13]. Briefly, air-dried capitula were reflux-extracted with 70% ethanol (1:20 g/mL) at 80 °C for 1 h. The crude extract was adsorbed onto HPD-300 macroporous resin (0.0625 g/mL) at 2 BV/h for 4 BV. After washing with water and sequential elution with 50% and 70% ethanol, the CE was obtained. LC-MS/MS analysis identified 74 phenolic compounds, of which the four major constituents—isookanin-7-*O-β*-D-glucoside, quercetin-7-*O-β*-D-glucoside, marein, and okanin—were quantitated (18.00%, 6.50%, 31.88%, and 2.58%, respectively). These four compounds represent the primary bioactive components of CE.

### 2.3. AI-Integrated Network Pharmacology and Affinity Screening

#### 2.3.1. Compound Prioritization and Target Identification

A composite scoring system was developed to prioritize 74 phenolic compounds from CE. Molecular descriptors (MW, logP, HBD, HBA, TPSA, rotatable bonds) were calculated with RDKit (V:2022.09.5); drug-likeness was assessed by Lipinski’s and Veber’s rules and structural alerts. An initial score (0–100) was computed as 100 minus total penalties. Absorption and toxicity were predicted using ADMETlab 2.0, applying penalties for high DILI/Ames risk. The final composite score was considered favorable, yielding 36 selected compounds. Targets of these 36 compounds were predicted via SwissTargetPrediction (probability > 0.1) and PharmMapper, merged to reviewed human UniProt entries. DN-related genes were identified from GEO dataset GSE30528 using limma (|log_2_FC| > 0.5, adjusted *p* < 0.05), resulting in 12,030 DEGs.

#### 2.3.2. AI-Based Affinity Prediction and Final Network Scoring

The overlap between compound-predicted targets and DN-associated DEGs yielded 2068 compound–target edges. A supervised machine learning framework was developed to predict compound–protein binding probability. Training data were obtained from the Binding DB database (https://www.bindingdb.org/; BindingDB_All_202406_tsv, accessed on 28 May 2024), consisting of 1,582,876 compound–target pairs. Five classifiers were compared: Logistic Regression (LR), Random Forest (RF), XGBoost, LightGBM, and a multi-layer perceptron (MLP). A 5-fold stratified cross-validation (random seed = 42) was performed on the entire dataset. For LR and MLP, features were standardized using a StandardScaler fitted on each training fold. All models were implemented with scikit-learn (v1.2, Python Software Foundation, Seattle, WA, USA), XGBoost (v1.7, DMLC community, Redmond, WA, USA), and LightGBM (v3.3, Microsoft Corporation, New York, NY, USA) using default hyperparameters, except MLP which had two hidden layers (512 and 256 neurons), ReLU activation, and early stopping (max 50 iterations). Performance was evaluated by area under the receiver operating characteristic curve (AUC-ROC) and average precision (AP). The best-performance MLP was applied to predict the binding probability (Predicted_Probability) for each of the 2068 edges in the CE–DN network.

The top-ranked targets and compounds were selected for pathway enrichment analysis (KEGG, details in Supplementary Table S2). This integrated approach combined classical network pharmacology with AI-driven affinity predictions, providing a systematic, quantitative framework for target identification.

### 2.4. Animals and Experimental Design

Specific pathogen-free (SPF) male Sprague Dawley rats (8 weeks old, 220 ± 20 g) were obtained from the Animal Center of Xinjiang Medical University (license No. SCXK (Xin) 2023-0001; use license No. SYXK (Xin) 2023-0004). All procedures were approved by the Institutional Laboratory Animal Ethics Committee of Xinjiang Medical University (Approval Code. IACUC-20211224-33, 24 December 2021) and followed the Guide for the Care and Use of Laboratory Animals (Ministry of Health, China) [16]. After 1 week of acclimatization, rats were randomly divided into a control group (*n* = 10) fed a standard diet (65% carbohydrate, 11% fat, 24% protein; 2.34 kcal/g) and a model group (*n* = 50) fed a high-fat diet (35% fat, 43.9% carbohydrate, 35.5% fat, 20.6% protein; 3.941 kcal/g) for 4 weeks. Diabetic nephropathy was induced by a single intraperitoneal injection of STZ (35 mg/kg, dissolved in ice-cold 0.01 M sodium citrate buffer, pH 4.4) after 12 h fasting; control rats received buffer only. Fasting blood glucose (FBG) was measured 3 days later via tail vein (Roche glucometer); rats with FBG between 16.7 and 33 mM were considered diabetic. At week 8, blood urea nitrogen (BUN) and serum creatinine (SCr) were measured to confirm renal impairment [17]. Successfully induced DN rats were randomized into five groups (*n* = 10/group): DN (vehicle), metformin (200 mg/kg/day, positive control, DN + ME200), and three CE treatment groups (150, 300, and 600 mg/kg/day, DN + CE150, DN + CE300, DN + CE600). Treatments were administered by oral gavage for 4 weeks. At week 12, rats were euthanized under pentobarbital sodium anesthesia; blood was collected from the abdominal aorta. Left kidneys were fixed in 10% neutral formalin for histology; right kidneys were snap-frozen for molecular analysis.

### 2.5. Biochemical Analysis

Urine was obtained from each rat and centrifuged under 3000 rpm for 10 min. The supernatant was analyzed for 24 h urinary albumin levels according to the protocol of urinary protein test kit. The blood samples were allowed to at room temperature for 3 h, followed by centrifugation at 3000 rpm for 15 min to obtain serum, which was prepared and stored at −80 °C until use. Serum levels of BUN, SCr, total cholesterol (TC), triglycerides (TG), insulin, levels of kidney AGEs, TNF-*α*, IL-1*β* and IL-6 were determined using corresponding assay kits according to their protocols.

### 2.6. Renal Histology Analysis

The left kidney tissue samples were fixed in 10% neutral paraformaldehyde, subjected to standard histological processing and embedded in paraffin. The sample was cut to 4 μm thick (RM2245, Leica Biosystems, Nussloch, Germany), stained with H&E, Periodic Acid–Schiff (PAS) and Masson–Fontana following standard protocols. Photographs were taken in a blinded fashion at random fields. Representative views of kidney sections were shown. The mesangial matrix index is the ratio of mesangial matrix area divided by the tuft area. The PAS and Masson–Fontana-positive areas were quantified in a blinded manner using ImageJ software (version 1.53, National Institutes of Health, Bethesda, MD, USA) with 10 random fields per kidney section [18].

### 2.7. Western Blotting

Right kidney tissues were homogenized in RIPA buffer (pH 7.4) containing protease inhibitors (1 M PNPP, 1 M NaF, 10 mM PMSF, 100 mM benzamidine, 100 mM DTT, 200 mM sodium orthovanadate). Protein concentration was determined by BCA assay (Pierce). Equal amounts (30 μg) were separated by 10% SDS-PAGE and transferred to PVDF membranes (Mini-PROTEAN Tetra System, Bio-Rad Laboratories, Hercules, CA, USA; Bio-Rad Laboratories, Hercules, CA, USA). Membranes were blocked with 5% BSA for 1 h and incubated overnight with primary antibodies, then with HRP-conjugated secondary antibodies. Protein bands were detected with enhanced chemiluminescence substrates (Thermo Fisher Scientific, Waltham, MA, USA) after washing. Blots were visualized and quantified by using a ChemiDoc system with ImageLab software (v4.1, Bio-Rad, CA). The level of protein expression in the liver was examined and normalized using *β*-actin as an internal control [19].

### 2.8. RNA Extraction and qPCR Analysis

Total RNA was extracted from the renal cortex (~10 mg) using TRIzol reagent. cDNA was synthesized using the FastQuant RT Kit (TIANGEN). qPCR was performed using 2× QuantiNova SYBR Green Master Mix on an ABI 7500 system (Applied Biosystems, Foster City, CA, USA). Relative gene expression was calculated using the 2^−ΔΔCt^ method with GAPDH as reference. Primer sequences are listed in Table 1.

### 2.9. Statistical Analysis

Differences were analyzed graphically using GraphPad Prism 6.0 software (Graph Pad, La Jolla, CA, USA). Ordinary one-way analysis of variance was used in the comparisons among different groups. Levene’s test was used before applying one-way ANOVA. The test indicated that the variances were homogeneous (*p* > 0.05 for all comparisons). Therefore, standard ANOVA was followed by Tukey’s post hoc test without any correction for unequal variances. Statistical results were expressed as the mean ± standard deviation (x ± SD). Differences with *p* < 0.05 were considered statistically significant.

## 3. Results and Discussion

### 3.1. AI-Integrated Screening Identifies Core Active Polyphenols and Key Targets of CE Against DN

**Figure 1:**
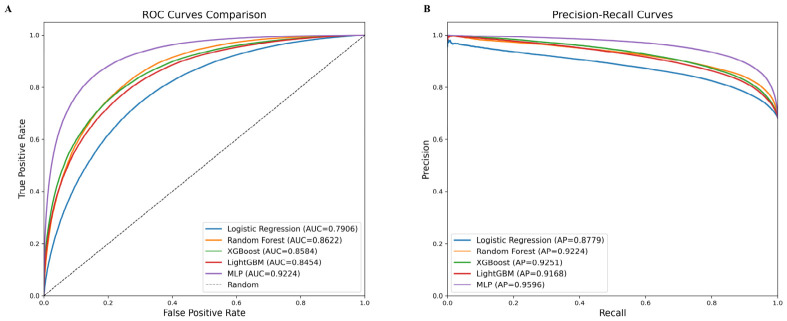
ROC and PR curves of different ML models. (**A**) ROC curve comparison. (**B**) Precision–recall curves (different colored lines represent Logistic Regression (LR), Random Forest (RF), XGBoost, LightGBM, and multi-layer perceptron (MLP), respectively. AUC-ROC and average precision (AP) values for each model are shown in the legend).

An integrated network pharmacology and machine learning framework was applied to systematically prioritize the active compounds and potential targets of CE against DN. A supervised classifier was trained on 3,165,752 compound–target pairs from Binding DB (v2024) to predict binding probability. Five algorithms were compared using 5-fold stratified cross-validation (Figure 1): Logistic Regression (LR), Random Forest (RF), XGBoost, LightGBM, and a multi-layer perceptron (MLP) [20,21]. The receiver operating characteristic (ROC) curve assesses the model’s ability to discriminate between positive and negative classes across all thresholds, while the precision–recall (PR) curve is particularly informative for imbalanced datasets, capturing the trade-off between precision and recall [22]. Among the five models, the MLP substantially outperformed all others, achieving a mean AUC-ROC of 0.9219 (SD = 0.0003) and a mean average precision (AP) of 0.9596 (SD = 0.0004). Despite requiring the longest training time, the MLP’s superior predictive accuracy justified its selection for downstream affinity prediction.

**Figure 2:**
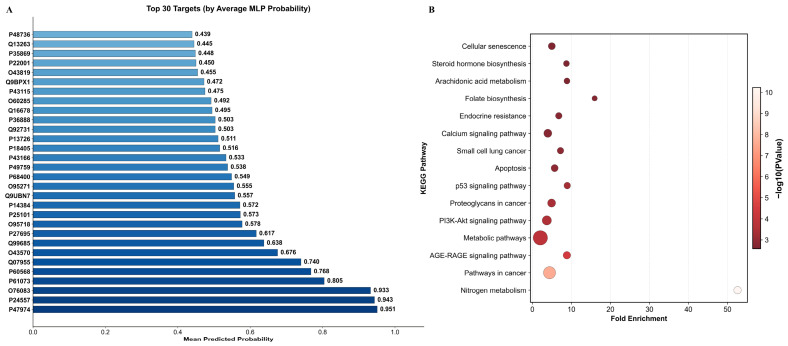
The top 30 targets with the highest probabilities and the KEGG pathway analysis. (**A**) Top 30 targets (by average MLP probability); (**B**) KEGG pathway.

The trained MLP was then applied to 2068 compound–target edges derived from the intersection of 36 prioritized CE polyphenols and 12,030 DN-associated DEGs. Compounds were ranked by their average predicted binding probability across all connected targets. The predicted probabilities of the 36 polyphenols ranged from 0.03 to 0.565. The five most active compounds were Compound **33** (7,8,3′,4′-Tetrahydroxyflavanone), Compound **15** (isoliquiritigenin), Compound **34** (3,7,8,2′,4′-Pentahydroxyflavone), Compound **29** (Butein-4′-O-glucoside), and Compound **13** (4-O-Caffeoyl quinic acid). Except for Compound **13**, the remaining four belong to the flavonoid or chalcone classes. The structures and complete ranking of all 36 compounds are provided in Supplementary Figures S1 and S2. On the target side, the top-ranked targets with the highest predicted binding probabilities included several proteins with established or emerging relevance to DN pathogenesis. Notably, inducible nitric oxide synthase (iNOS/NOS2, UniProt P35228), a key mediator of oxidative stress in diabetic kidneys, was among the high-confidence targets [23]. In addition, the screen identified several nuclear receptors and transcriptional regulators—including the estrogen-related receptors (ESRRA, P11474; ESRRB, O95718), estrogen receptors (ESR1, P03372; ESR2, Q92731), aryl hydrocarbon receptor (AHR, P35869), and tripartite motif-containing **28** (TRIM28, Q13263)—which have been increasingly implicated in metabolic regulation and renal protection. Among other notable candidates, phosphoinositide-3-kinase catalytic subunit gamma (PIK3CG, P48736) was linked to the PI3K-Akt signaling pathway, while steroid 5α-reductase **1** (SRD5A1, P18405) and phosphodiesterase **9**A (PDE9A, O76083) represent less-characterized targets that may offer novel therapeutic opportunities. These AI-prioritized targets provided a rational basis for the selective experimental validation of key DN-associated pathways reported in subsequent sections, including NF*κ*B, Nrf2/HO-1, TGF*β*/Smad, and PPAR*γ* signaling (Figure 2A and Table S2).

The average predicted probabilities for the top 100 targets (Table S2) were concentrated between 0.19 and 0.95, indicating strong and consistent prediction confidence. To elucidate the biological processes that underlie these predicted CE–DN interactions, KEGG pathway enrichment analysis was performed on the top 100 targets, revealing 15 significantly enriched pathways (adjusted *p* < 0.05) as visualized in Figure 2B and Table S3. Among these, the most prominent pathways included “Metabolic pathways”, “Pathways in cancer”, “PI3K-Akt signaling pathway”, “Calcium signaling pathway”, “Cellular senescence”, and “Apoptosis”. Notably, several pathways directly linked to DN pathogenesis—such as the PI3K-Akt pathway (involved in podocyte injury and mesangial expansion), calcium signaling (mediating vasoconstriction and oxidative stress), and Metabolic pathways (contributing to glomerular endothelial dysfunction)—were significantly enriched. These findings suggest that CE may exert its renoprotective effects by modulating a network of signaling pathways that converge on inflammation, fibrosis, and metabolic dysregulation.

These integrative findings indicate that the AI-prioritized targets not only recapitulate the core pathological machinery of DN but also extend it to less-characterized regulatory nodes. The simultaneous enrichment of PI3K-Akt, calcium signaling, and metabolic pathways suggests that CE polyphenols may act on a multi-target network that converges on inflammation, fibrosis, and metabolic dysregulation—processes central to DN progression. The identification of both canonical targets (e.g., TGF*β*1, PPAR*γ*) and newly associated proteins (e.g., SRD5A1, PDE9A, ZFP36L2) underscores the value of combining machine learning with network pharmacology to uncover the full spectrum of bioactive ingredients. Together, these results provided a rational basis for the selective experimental validation of key DN-associated pathways reported in the subsequent sections, including NF*κ*B, Nrf2/HO-1, TGF*β*/Smad, and PPAR*γ* signaling.

### 3.2. CE Improves Metabolic Parameters and Renal Function in DN Rats

**Figure 3:**
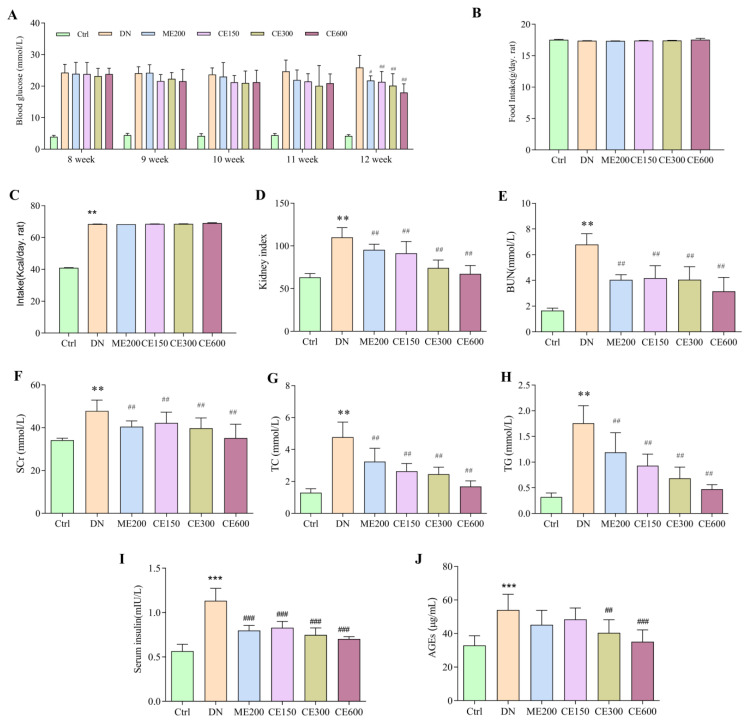
Detection of blood glucose level and biochemical parameters of kidney functions. (**A**) Effects of CE on blood glucose; (**B**,**C**) food and energy intake; (**D**) kidney index; (**E**) blood urea nitrogen level in serum of different groups; (**F**) creatinine level in serum of different groups; (**G**) TC level in serum of different groups; (**H**) TG level in serum of different groups; (**I**) insulin level in serum of different groups; (**J**) AGE level in the renal tissue of different groups. * *p* < 0.05, ** *p* < 0.01, *** *p* < 0.001 as compared to Ctrl group; # *p* < 0.05, ## *p* < 0.01, ### *p* < 0.001 as compared to DN group.

CE significantly reduced blood glucose levels in DN rats after 12 weeks (Figure 3A). Although food intake did not differ among groups (Figure 3B), energy intake was higher in rats fed the high-fat diet (Figure 3C). DN rats exhibited kidney hypertrophy, as indicated by increased kidney weight and kidney index, both of which were significantly attenuated by CE treatment (Figure 3D). Furthermore, serum markers of kidney injury including BUN and SCr were significantly elevated in DN rats, reflecting impaired renal function. CE treatment effectively reduced these markers at all tested doses (Figure 3E,F). In terms of lipid metabolism, CE exerted dose-dependent effects at all tested reductions in serum TC and TG in DN rats fed a high-fat diet (Figure 3G,H), indicating beneficial regulation of dyslipidemia. Additionally, while serum insulin levels were significantly increased in the DN group, CE treatment substantially lowered insulin concentrations at all tested doses (Figure 3I). CE also significantly lowered renal AGEs accumulation, which were markedly elevated in DN rats. This reduction was effective at all tested doses, with significantly greater effects observed at 300 and 600 mg/kg doses compared to 150 mg/kg (Figure 3J). Collectively, these results suggest that CE improves metabolic parameters and renal function in DN rats.

In the DN rat model, CE treatment significantly ameliorated hyperglycemia, insulin resistance, and dyslipidemia, consistent with the predicted modulation of metabolic pathways. The observed reductions in serum creatinine, blood urea nitrogen, and renal AGEs accumulation further indicated that CE protects renal function, likely through the regulation of pathways enriched in the computational screen, such as glucose and lipid metabolism [24]. Natural products have been reported to lower the AGEs in diabetic rats by reducing oxidative stress and hyperglycemia [25,26]. These results align with previous reports that dietary polyphenols can improve metabolic parameters in diabetic models [27,28] and extend these findings by linking the metabolic improvements to specific compound–target pairs identified through AI-guided prioritization.

### 3.3. CE Ameliorates Renal Histopathology, Fibrosis, and Inflammation in DN Rats

**Figure 4:**
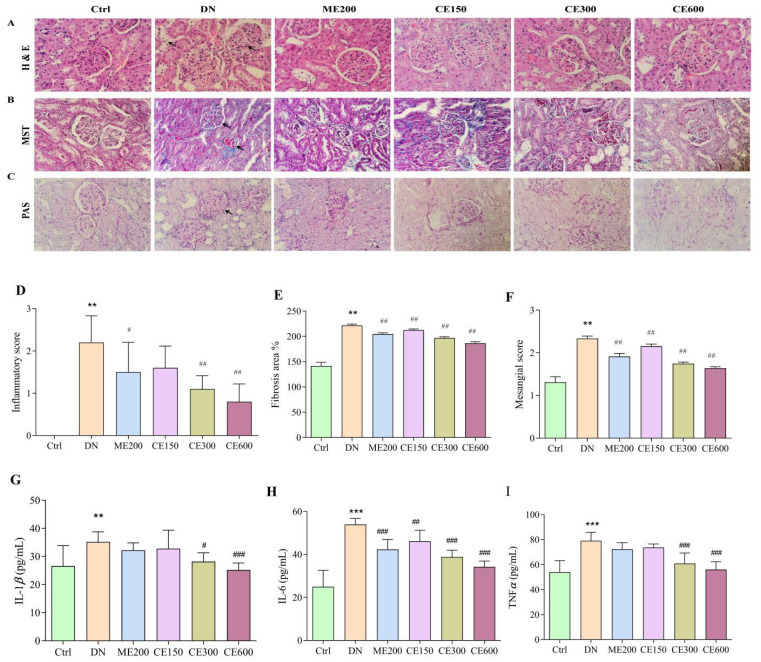
Effect of CE on kidney pathology. (**A**) HE staining, control group showing normal kidney structure with glomerulus and proximal tubules; disease group (DN) showing glomerular degeneration (asterisk), swollen and damaged tubules (arrows). (**B**) MST-stained sections in different groups showing the fibrous tissue deposition (fibrous tissue deposition areas are stained blue). (**C**) PAS-stained sections in different groups showing deposition of the mesangial matrix. (**D**–**F**) Graphical representation of relative degenerative changes, glomerular size and fibrous tissue deposition in various groups. (**G**–**I**) The pro-inflammatory cytokines IL-1*β*, TNF-*α* and IL-6 and their tissue levels. * *p* < 0.05, ** *p* < 0.01, *** *p* < 0.001 as compared to Ctrl group; # *p* < 0.05, ## *p* < 0.01, ### *p* < 0.001 as compared to DN group.

Histopathological analysis revealed normal renal architecture in control rats, whereas DN induced extensive pathological alterations, including glomerular degeneration with heightened eosinophilia, urinary space expansion, and proximal tubular injury featuring cellular swelling and brush border loss (Figure 4A). CE treatment significantly alleviated these degenerative alterations at all doses, with the most prominent renoprotective effect at the highest dose (CE600). DN rats also exhibited marked glomerular hypertrophy compared to controls, which was effectively prevented by CE administration (Figure 4D). Consistent with these findings, MST staining revealed substantial fibrous tissue deposition within the renal parenchyma in DN rats, indicative of fibrosis progression (Figure 4B). CE treatment significantly reduced fibrous tissue accumulation at all doses (Figure 4E). Additionally, PAS staining showed increased mesangial matrix expansion in DN rats, reflecting mesangial proliferation and podocyte injury (Figure 4C). CE treatment markedly attenuated this mesangial proliferation and reduced PAS-positive areas in glomeruli (Figure 4F). Renal inflammation is a key driver of DN pathogenesis [29]. Pro-inflammatory cytokines, including TNF-*α*, IL-1*β*, and IL-6, were significantly elevated in DN kidneys but were notably suppressed by CE treatment at all doses (Figure 4G–I), which correlated well with the improved histological outcomes [30,31]. Together, these results suggested that CE effectively alleviated key pathological hallmarks of diabetic nephropathy, including glomerular and tubular damage, renal fibrosis, mesangial proliferation, podocyte injury, and local inflammatory responses. These results further support the renoprotective effects of CE.

### 3.4. CE Modulates Renal Inflammation and Oxidative Stress Signaling Pathways in DN

**Figure 5:**
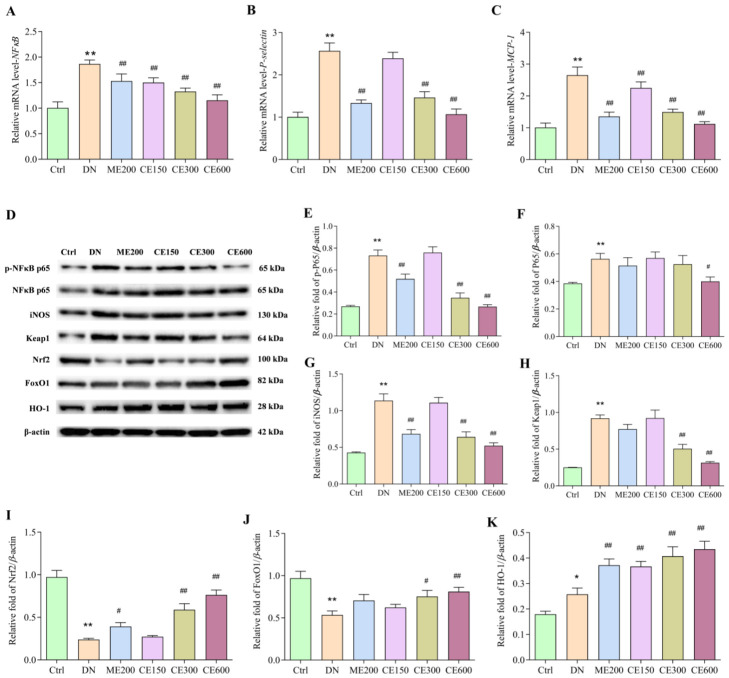
The effect of CE on levels of various inflammatory markers. (**A**–**C**) The mRNA levels of NF*κ*B, P-selectin and MCP-1 were estimated by qPCR in the kidney tissue of different groups; (**D**–**K**) The expression levels of p-NF*κ*Bp65, NF*κ*Bp65, iNOS, Keap1, Nrf2, FoxO1 and HO-1 were estimated by Western blot in the kidney tissue of different groups. * *p* < 0.05, ** *p* < 0.01, *** *p* < 0.001 as compared to Ctrl group; # *p* < 0.05, ## *p* < 0.01, ### *p* < 0.001 as compared to DN group.

Inflammation and oxidative stress are interconnected drivers of DN pathogenesis [32]. In this study, the disease control group showed significantly elevated expression of the pro-inflammatory transcription factor NF*κ*B in kidney tissue when compared to the controls (*p* < 0.05). CE treatment markedly suppressed NF*κ*B expression at all doses (*p* < 0.05), indicating its potent anti-inflammatory properties (Figure 5A,D–F). Furthermore, the gene expressions of inflammatory mediators MCP-1 and P-selectin, which contribute to leukocyte recruitment and adhesion in kidney inflammation, were also significantly reduced by CE treatment in a dose-dependent manner (Figure 5B,C).

The iNOS/Nrf2/HO-1 signaling pathway plays a pivotal role in modulating cellular redox balance and inflammatory responses. In DN rats, CE treatment dose-dependently decreased iNOS and Keap1 expression, both of which are indicators of oxidative stress and negative regulators of antioxidant responses (*p* < 0.05). Meanwhile, the expression levels of Nrf2 and its downstream target HO-1, which are essential antioxidants, were significantly elevated following CE administration in a dose-responsive manner (*p* < 0.05) (Figure 5D,I–K). Moreover, CE treatment reversed the diabetic conditions induced suppression of FoxO1 protein, a transcription factor involved in cellular stress resistance and metabolic regulation (Figure 5D,G). These findings suggested that CE exerts renoprotective effects through the coordinated suppression of inflammatory pathways and the enhancement of antioxidant defenses, highlighting its potential as a functional food component for alleviating inflammation and oxidative damage in diabetic nephropathy.

Inflammation and oxidative stress are interconnected drivers of DN pathogenesis [33]. CE treatment suppressed the NF*κ*B pathway, as reflected by decreased phosphorylation of NF*κ*Bp65 and reduced expression of its downstream targets, including MCP-1, P-selectin, IL-1*β*, IL-6, and TNF-*α*. The Nrf2/HO-1/Keap1 signaling pathway regulates the expression of antioxidant genes and plays a protective role in DN animal models [34,35]. It has been proven that anthocyanins derived from *C. tinctoria* exhibit strong antioxidant properties, which may protect MIN6 cells against oxidative damage by promoting the translocation of Nrf2 into the nucleus, thereby efficiently reducing cellular ROS production and cell apoptosis [36]. Concurrently, CE activated the Nrf2/HO-1/FoxO1 antioxidant axis, leading to enhanced cellular defense against oxidative stress. These dual actions—inhibiting pro-inflammatory signaling while boosting antioxidant capacity—are consistent with the enriched “Cellular senescence” and “Apoptosis” pathways identified in the KEGG analysis.

### 3.5. CE Attenuates Renal Fibrosis and Podocyte Injury via TGFβ/Smad Pathway in DN

**Figure 6:**
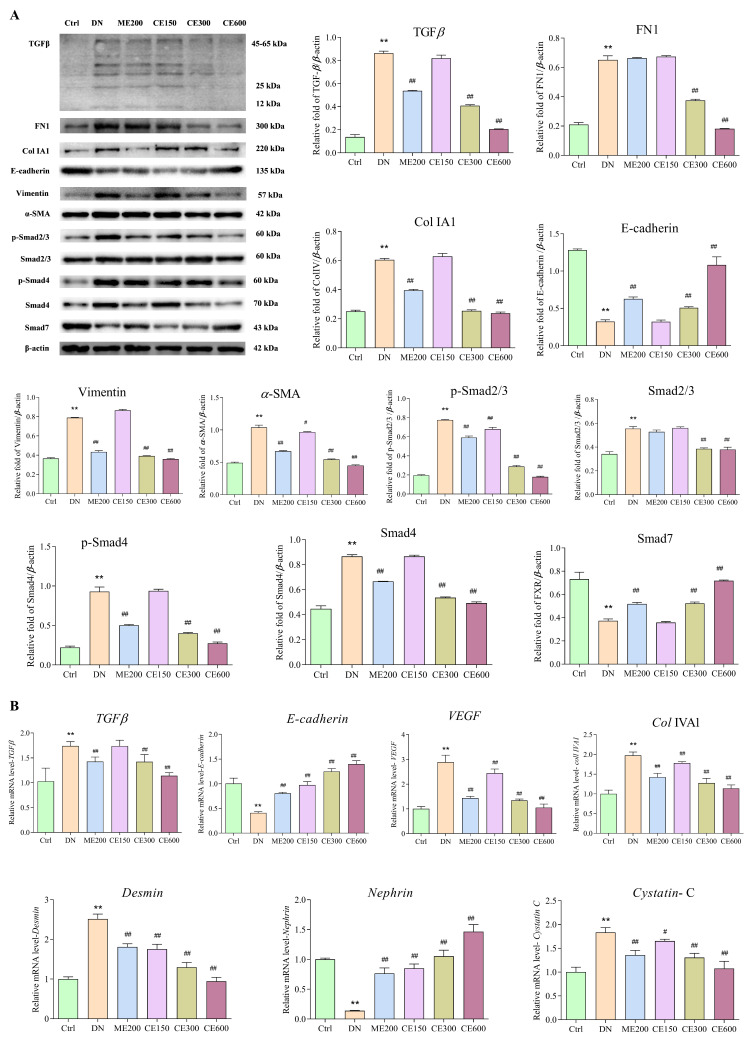
Effect of CE on renal fibrous tissue deposition markers. (**A**) The expression of TGF*β*, FN1, ColIA1, E-cadherin, Vimentin, *α*-SMA, p-Smad2/3, Smad2/3, p-Smad4, Smad4 and Smad7 were estimated by Western blot in the kidney tissue of different groups. (**B**) The mRNA levels of TGF*β*, E-cadherin, VEGF, ColIVA1, Desmin, Nepherin and Cystatin-C were estimated by qPCR in the kidney tissue of different groups. * *p* < 0.05, ** *p* < 0.01, *** *p* < 0.001 as compared to Ctrl group; # *p* < 0.05, ## *p* < 0.01, ### *p* < 0.001 as compared to DN group.

DN resulted in substantial renal fibrosis, as indicated by an increase in the expression of transforming growth factor beta (TGF*β*) at both the protein and mRNA levels (*p* < 0.05). This effect was significantly attenuated by CE treatment at doses of 150, 300, and 600 mg/kg/day (Figure 6A,B). In addition, DN rats exhibited elevated expression of fibrosis-associated markers including fibronectin 1 (FN1), collagen IA1 (ColIA1), vimentin, *α*-smooth muscle actin (α-SMA), phosphorylated Smad2/3 (p-Smad2/3), total Smad2/3, phosphorylated Smad4 (p-Smad4), and Smad4. CE administration significantly downregulated these profibrotic proteins after 4 weeks of treatment (Figure 6A). Conversely, the expression levels of the epithelial marker E-cadherin and the inhibitory Smad7, both of which are critical for preserving renal structural integrity, were significantly enhanced following CE supplementation (*p* < 0.05) (Figure 6A,B).

The TGF*β*/Smad signaling pathway results in integrity destruction of glomerular filtration barrier and the occurrence of proteinuria, thereafter, triggering the occurrence of DN [37]. The results demonstrated that CE suppressed the TGF*β*/Smad signaling cascade, as evidenced by decreased expression of TGF*β*1, p-Smad2/3, Smad4, and downstream fibrosis markers (FN1, Col1A1, *α*-SMA, vimentin), while restoring E-cadherin and Smad7 levels. This anti-fibrotic effect was predicted by the network pharmacology screen, which identified TGF*β*1 as one of the top-ranked targets with a high predicted binding probability. The podocyte injury can be detected by Nephrin, Desmin and Cystatin-C expressions, resulting glomerular structure and function alteration. Nephrin is crucial for maintaining the slit diaphragm, glomerular ultrafiltration and podocyte function [38], whereas Desmin is an important cytoskeletal protein [39]. Furthermore, CE preserved podocyte integrity by upregulating nephrin and downregulating desmin, VEGF, and Col4A1, thereby protecting the glomerular filtration barrier. The involvement of the VEGF pathway in podocyte injury has been well documented [40], and our findings provide mechanistic evidence supporting its regulation by CE. Elevated AGE levels increase the expression of AGE receptors on the cells, which in turn leads to oxidative stress, activation of NF*κ*B, and the secretion of various growth factors (TGF*β* and connective tissue growth factor) and cytokines [41,42]. These processes contribute to diabetic vascular complications, renal inflammation and fibrosis [43,44].

### 3.6. CE Modulates PPARγ/FXR/STAT3 Signaling Pathway and Inhibits Renal Cell Apoptosis in DN

**Figure 7:**
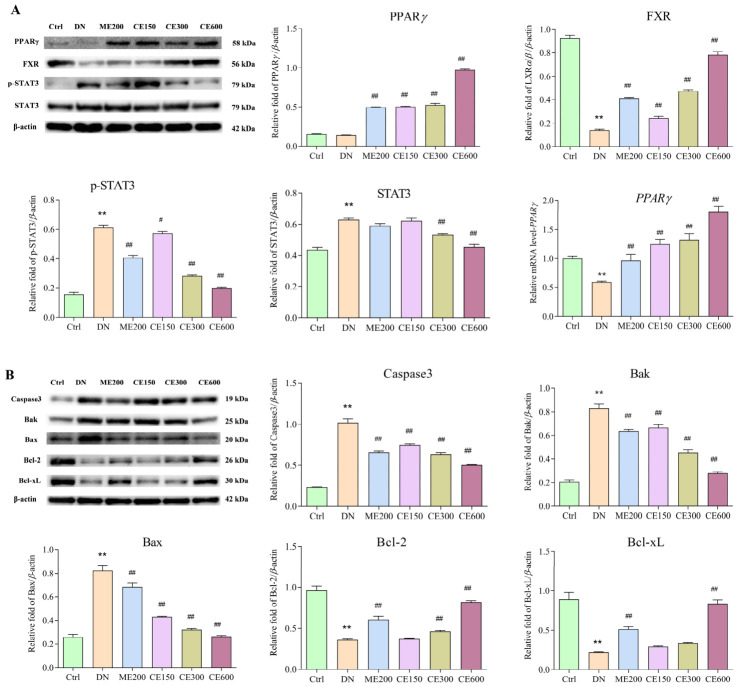
Effect of CE on PPAR*γ* and apoptosis pathways. (**A**) The expression of PPAR*γ*, LXR*α*/*β*, p-STAT3 and STAT3 in the kidney tissue of different groups. (**B**) The expression of Caspase3, Bak, Bax, Bcl-2 and Bcl-xL were estimated by Western blot in the kidney tissue of different groups. * *p* < 0.05, ** *p* < 0.01, *** *p* < 0.001 as compared to Ctrl group; # *p* < 0.05, ## *p* < 0.01, ### *p* < 0.001 as compared to DN group.

In DN rats, renal expression of PPAR*γ* was significantly reduced compared to controls. CE administration at doses of 150, 300, and 600 mg/kg significantly upregulated both the protein and mRNA levels of PPAR*γ* in kidney tissue (*p* < 0.05) (Figure 7A). Similarly, the expression of Farnesoid X receptor (FXR), which was notably downregulated in DN rats, was also restored by CE treatment. Concurrently, the phosphorylated and total levels of signal transducer and activator of transcription 3 (p-STAT3 and STAT3), which were elevated in DN, were significantly suppressed following CE administration, indicating modulation of this pro-inflammatory and pro-fibrotic signaling pathway (Figure 7A). Renal cell apoptosis, a key contributor to diabetic kidney injury, was assessed by examining apoptosis-related proteins. Caspase-3 protein levels were markedly increased in DN rats but were significantly reduced by CE treatment at all doses (*p* < 0.05) (Figure 7B). Similarly, pro-apoptotic markers Bak and Bax were found to be elevated in diabetic kidneys and were effectively downregulated by CE (*p* < 0.05). Conversely, anti-apoptotic proteins Bcl-2 and Bcl-xL, which were decreased in DN, showed significant restoration upon CE treatment (*p* < 0.05).

The observed reduction in renal apoptosis, as evidenced by decreased caspase-3, Bak, and Bax, along with restored Bcl-2 and Bcl-xL, further supports the protective role of CE against DN-associated renal cell death. Caspase 3 activation measurements showed that *C. tinctoria* flowering tops extracts, as well as marein and flavanomarein, significantly inhibit apoptosis on tBHP and cytokine-induced cell injury in pancreatic MIN6 cells [45].

PPAR*γ*, as a nuclear receptor with important metabolic functions, has been shown to regulate inflammation, lipid metabolism, and insulin sensitivity, thereby improving renal outcomes in DN [46]. Critically, PPAR*γ* activation inhibits NF*κ*B-mediated inflammatory gene transcription, highlighting its important role in mediating the connection between nutrition and metabolic homeostasis [47]. CE modulated the PPAR*γ*/FXR/STAT3 signaling axis, which has been implicated in lipid metabolism, inflammation, and fibrosis in DN [48,49,50], which in turn inhibited the downregulation of key genes involved in fibrosis such as TGF*β*1, collagen, and fibronectin [51]. The upregulation of PPAR*γ* and FXR, coupled with the inhibition of STAT3 phosphorylation, suggests that CE may alleviate lipotoxicity-induced renal injury by restoring lipid homeostasis and suppressing inflammatory cascades.

It has been proven that inhibition of STAT3 phosphorylation involved in alleviating cellular senescence and apoptosis in DN [52]. This is particularly relevant given that dyslipidemia is a major contributor to DN progression [53], and PPAR*γ* agonists have demonstrated renoprotective effects in clinical and preclinical studies [54].

**Figure 8:**
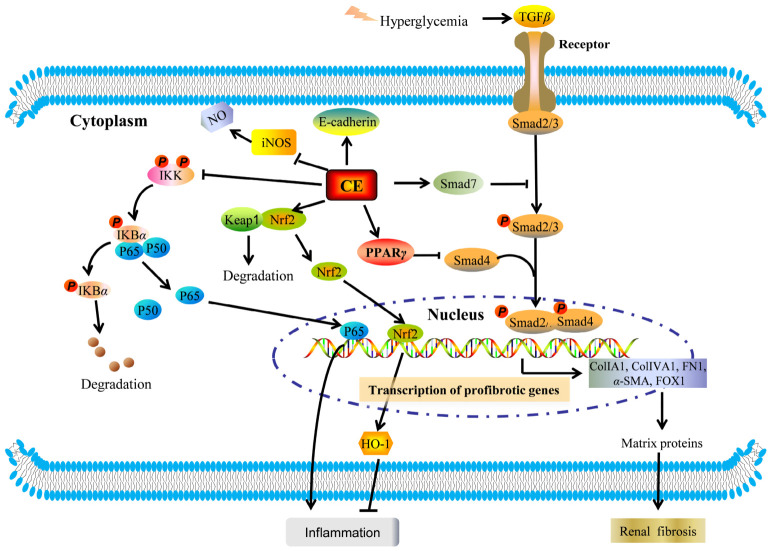
A schematic representation of the anti-fibrotic effect of CE in the inhibition of kidney TGF*β* pathway. Activation of PPAR*γ* by CE treatment promotes the translocation of Nrf2 to the nucleus, which subsequently reduced oxidative stress and elevating Smad7 protein levels, thereby inhibiting Smad2/3 phosphorylation to block TGF*β* signaling. These consecutive events result in fibrotic factors suppression and ultimately attenuates hyperglycemia-induced renal inflammation and fibrosis.

This study demonstrates that *C. tinctoria* extract, through its rich polyphenolic composition, exerts renoprotective effects in diabetic nephropathy by modulating a multi-target network involving PI3K-Akt, TGF*β*/Smad, NF*κ*B, Nrf2/HO-1/FoxO1, and PPAR*γ*/FXR/STAT3 signaling pathways (Figure 8). Nutritional interventions targeting these pathways can be effective in preserving renal function [5]. The integration of AI-driven network pharmacology with experimental validation provides a systematic and quantitative framework for identifying active compounds and their mechanisms of action in complex diseases. These findings support the potential of CE as a functional food ingredient for the prevention and management of diabetic nephropathy.

Several limitations of this study should be acknowledged. First, while the MLP model achieved high predictive accuracy, the training data were derived from the Binding DB database, which may not fully represent the chemical diversity of natural products. Future studies could incorporate natural product-specific training sets to improve model generalizability. Second, although the top-ranked compounds and targets were validated in vivo, the individual contribution of each compound remains to be elucidated. Further studies using purified compounds or combinatorial approaches are warranted. Third, the present study employed a single DN rat model (high-fat diet plus low-dose STZ); additional models, such as db/db mice or STZ-induced type 1 diabetic models, would strengthen the generalizability of the findings.

## 4. Conclusions

In conclusion, this study integrated AI-driven network pharmacology with in vivo experimental validation to elucidate the mechanism of *C. tinctoria* extract (CE) against diabetic nephropathy (DN). The computational screening identified 36 active polyphenols and 100 high-confidence targets, with subsequent pathway analysis pointing to PI3K-Akt, calcium signaling, and metabolic pathways as key hubs. In vivo experiments confirmed that CE significantly improved glucose and lipid metabolism, reduced renal dysfunction markers (SCr, BUN), and alleviated renal fibrosis and inflammation. These findings suggest that CE, as a polyphenol-rich natural product, holds promise as a functional dietary supplement for the prevention and management of DN and associated metabolic disorders. Future studies should focus on clinical translation, including dose-optimization trials in humans and the investigation of synergistic effects among the top-ranked active compounds. Additionally, incorporating single-cell transcriptomics and proteomics could further refine the target network and uncover cell-type-specific mechanisms underlying CE’s renoprotective effects.

## Figures and Tables

**Table 1:** Primer sequences.

Gene Name	Sequence
*Cystatin-C*	ACTAACTGTCCTTTCCACGA
	AGGGTAGGGGAACAAGTAAG
*VEGF*	GCAGACTATTCAACGGACTC
	CGAAGTAATTTGAGGGAGTG
*P-selectin*	GGTCACTGGTCAGATGCTAT
	TTTCTTTCTGAGACGCTTTC
*MCP-1*	TGTCCCAAAGAAGCTGTAGT
	AGTTCACATTCAAAGGTGCT
*Col IV4A* *1*	GGTGTGAACTAACTGGCTTC
	GTAAGACAGCTGGAAAGGTG
*E-Cadherin*	GAAGACAGAAACGAGACTGG
	GTCTCCCTCTCAATGATGAA
*NFκB*	ATGACATTGAGGTTCGTTTC
	TACTTCCTCCTTGTCTTCCA
*PPARγ*	CATGACCAGGGAGTTCCTCAA
	AGCAAACTCAAACTTAGGCTCCAT
*Nephrin*	CTCAGTGATGACGCAGAGTA
	GACACACAGGTGACCACATA
*Desmin*	GTCCACACCAAAAAGACAGT
	AGACCACAAAGAGGTGATTG
*TGFβ*	CTGAACCAAGGAGACGGAAT
	GACTGATCCCATTGATTTCCA
*GAPDH*	ACAGCAACAGGGTGGTGGAC
	TTTGAGGGTGCAGCGAACTT

## Data Availability

The original contributions presented in this study are included in the article/Supplementary Materials. Further inquiries can be directed to the corresponding author.

## Supplementary Materials

The following supporting information can be downloaded at: https://www.mdpi.com/article/10.3390/foods15132257/s1, Table S1. List of primary and secondary antibodies; Table S2. The key 100 targets predicted by MLP; Table S3. KEGG signal pathway enrichment analysis; Figure S1. The structure of active compounds; Figure S2. The complete ranking of all 36 compounds.

## Abbreviations

CE	*Coreopsis tinctoria* Extract
DN	Diabetic nephropathy
ML	Machine learning
PI3K	Phosphoinositide 3-Kinase
Akt	Protein Kinase B
NFκB/p65	Nuclear factor NF-kappa-B2
iNOS	Inducible nitric oxide synthase
Keap1	Kelch-like ECH-associated protein 1
GAPDH	Glyceraldehyde-3-phosphate dehydrogenase
COL1A1	Collagen type I alpha 1 chain
*α*-SMA	Alpha-smooth muscle actin
Caspase3	Cysteine-aspartic acid specific protease-3
Bak	BCL2-antagonist/killer 1
STAT3	Signal transducer and activator of transcription 3
FXR	Farnesoid X receptor
TGF*β*	Transforming growth factor beta
Smad	Sma- and mad-related proteins
PPAR*γ*	Peroxisome proliferator-activated receptor gamma
Nrf2	Nuclear factor erythroid 2-related factor 2
HO-1	Hemeoxygenase-1
FoxO1	Forkhead box protein O1
Bcl-2	B-cell lymphoma/leukemia-2
Bcl-xL	B-cell lymphoma 2-like protein 1
Bax	Bcl-2-associated X protein
STZ	Streptozotocin
TNF-*α*	Tumor necrosis factor-*α*
IL-1*β*	Interleukin-1*β*
IL-6	Interleukin-6
AGEs	Advanced glycation end products
FBG	Fasting blood glucose
SCr	serum creatinine
BUN	Blood urea nitrogen
TG	Total cholesterol
TC	Total cholesterol
HE	Hematoxylin–eosin
PAS	Periodic acid–Schiff
PNPP	p-Nitrophenyl phosphate
NaF	Sodium fluoride
PMSF	Phenylmethanesulfonyl fluoride
DTT	DL-dithiothreitol
BCA	Bicinchoninic acid assay
VEGF	Vascular endothelial growth factor
MCP-1	Monocyte chemoattractant protein1/(C-C motif) ligand 2
Col IV4A1	Alpha-1 type IV collagen
E-Cadherin	Cadherin-1/Epithelial cadherin
