# Supplementary material for: AI-Integrated Multi-Target Validation of Coreopsis tinctoria Polyphenols as a Functional Food Ingredient Against Diabetic Nephropathy
Source: Foods. 2026 Jun 23;15(13):2257. doi: 10.3390/foods15132257 (PMC13361528; doi:10.3390/foods15132257)
Supplement: Supplementary file 1 [file foods-15-02257-s001.zip › foods-4369682-supplementary.pdf]

AI-Integrated Multi-Target Validation of *Coreopsis tinctoria* Polyphenols as a Functional Food Ingredient Against Diabetic Nephropathy

Dilinare Abdurehman [1,2], Xueying Lu [1,2], Yindengzhi Guoruoluo [1], Geyu Liu [1], Jun Li [1,2], Tao Wu [1,2], Xuelei Xin [1,2] and Haji Akber Aisa [1,2,3,*]

[1] State Key Laboratory Basis of Xinjiang Indigenous Medicinal Plants Resource Utilization and Key Laboratory of Plants Resources and Chemistry of Arid Zone, Xinjiang Technical Institute of Physics and Chemistry, Chinese Academy of Sciences, Urumqi 830011, China; dilinare@ms.xjb.ac.cn (D.A.); xueyinglu@ms.xjb.ac.cn (X.L.); yindz@ms.xjb.ac.cn (Y.G.); liugy@ms.xjb.ac.cn (G.L.); lijun702@ms.xjb.ac.cn (J.L.); wutao@ms.xjb.ac.cn (T.W.); xinxl@ms.xjb.ac.cn (X.X.)

[2] University of Chinese Academy of Sciences, Beijing 100049, China

[3] Institute of Traditional Chinese Medicine, Xinjiang Medical University, Urumqi 830054, China

* Correspondence: haji@ms.xjb.ac.cn

Table S1. List of primary and secondary antibodies

Antibody Name	Product Number	Supplier
FN1	#268365	
TGFβ	#3711	
ColIA1	#72026	
E-cadherin	#14472s	
α-SMA	#19245S	
Vimentin	#5741	
p-Smad2/3	#8828S	
Smad2/3	#8685T	
Smad4	#46535	
FXR	#4713	
p-STAT3	#9131S	Cell Signaling Technology (CST)
NFκB p65	#8242s	
p-NFκBp65	#3033S	
Nrf2	#12721s	
Keap1	#4678	
FoxO1	#2880	
HO-1	#5853S	
Cleaved Caspase-3	#9664	
Bax	#2772S	
Bak	#3814S	
Bcl-2	#2870S	
Bcl-xL	#2764S	
PPARγ	#AB209350	Abcam
STAT3	#AB31370	
LXRα/β	sc-377260	Santa Cruz
Smad7	sc-365846	
Phospho-Smad4	#AF8316	Affinity
iNOS	#AF0199	
Anti-rabbit IgG	BA1054	BOSTER
Anti-mouse IgG	BA1050	

Table S2. The key 100 targets predicted by MLP.

Target	mean_prob	max_prob	count_compounds
P47974	0.9509233	0.9509233	1
P24557	0.94345635	0.94345635	1
O76083	0.93260465	0.9336475	2
P61073	0.80454594	0.86142284	2
P60568	0.767768947	0.995167	5
Q07955	0.74030286	0.74030286	1
O43570	0.6757399	0.9991596	28
Q99685	0.6378453	0.6378453	1
P27695	0.616815855	0.8314288	4
O95718	0.577710948	0.73898935	5
P25101	0.5728393	0.68152255	5
P14384	0.57175314	0.57175314	1
Q9UBN7	0.5573682	0.5573682	1
O95271	0.554628013	0.94185776	10
P68400	0.548546717	0.78512937	10
P49759	0.53779155	0.972741	4
P43166	0.533157957	0.9862982	24
P18405	0.51595459	0.9325278	6
P13726	0.511394369	0.84937364	7
Q92731	0.503363442	0.9910909	19
P36888	0.503355669	0.9708274	10
Q16678	0.494782586	0.9994271	20
O60285	0.492130302	0.98057866	9
P43115	0.47513968	0.47513968	2
Q9BPX1	0.472460325	0.5082986	2
O43819	0.45481005	0.6534286	4
P22001	0.450269958	0.9575033	4
P35869	0.448452295	0.8796226	10
Q13263	0.44485012	0.44485012	1
P48736	0.439472193	0.5939985	4
P35228	0.428574413	0.65760356	3
Q9H2K2	0.420819578	0.8880441	10
P11474	0.395430805	0.80869853	13
P09917	0.395293502	0.9890794	14
P09874	0.386846618	0.95324236	6
P10144	0.38459325	0.38459325	1
Q16875	0.376873657	0.81664926	7
Q9ULX7	0.369581235	0.94047785	11
P03372	0.369329993	0.92950875	17
P16050	0.366942216	0.9660026	9

P05164	0.363412018	0.85998213	9
P14635	0.363324367	0.5463062	3
Q9NPH5	0.362635278	0.95633686	16
P22083	0.362484723	0.7901132	3
O60218	0.35577074	0.88694954	11
Q00534	0.334417656	0.7687358	10
P00918	0.330381839	0.971262	28
P04278	0.316517826	0.84042317	8
P43235	0.31509092	0.31509092	1
P55735	0.312097613	0.8219804	4
P06493	0.310340109	0.8284228	11
O95067	0.308914074	0.87435627	9
P60953	0.3082476	0.3082476	1
O75582	0.3056711	0.3056711	1
P28845	0.3050641	0.3050641	1
Q13627	0.301410059	0.4422439	5
P37059	0.299891978	0.71064484	16
P16152	0.299077721	0.91964144	12
Q08499	0.296807399	0.6308031	5
P35968	0.296052282	0.9144396	13
P22748	0.292247037	0.90935796	23
Q00535	0.292042406	0.7464619	14
P41594	0.288402306	0.42434698	7
P08253	0.288280638	0.8114454	19
Q13133	0.2882175	0.2882175	1
P35218	0.286941625	0.8652239	20
P42338	0.280763325	0.5537766	2
Q9HC97	0.271462304	0.76001775	8
Q16790	0.266580348	0.8901709	20
Q12791	0.265234613	0.6201449	3
O15530	0.263134613	0.32093045	3
P11309	0.262512491	0.716984	11
P53355	0.260702872	0.90729773	9
P24864	0.25858137	0.25858137	1
Q96AP0	0.2582428	0.2582428	1
P27487	0.25482762	0.25482762	1
P39900	0.252797239	0.79463524	23
P01375	0.252238142	0.59858716	5
Q9Y463	0.248970454	0.6293785	4
P30530	0.248548764	0.9247776	10
P31639	0.246134546	0.7921699	12
P00734	0.244766244	0.90777713	10

P10415	0.243804162	0.35101986	5
Q13554	0.242958799	0.8195175	9
P43405	0.238648178	0.9574137	11
Q15746	0.23559094	0.7387288	4
P15907	0.235565365	0.4371973	4
Q9UM73	0.233729531	0.9808292	11
Q15078	0.227783667	0.808366	5
P15121	0.224694659	0.9765861	29
P28907	0.215199293	0.7090426	13
Q15835	0.21121071	0.21121071	1
P42330	0.208172075	0.82663316	17
O14965	0.205320309	0.40152732	2
Q05397	0.204693407	0.9439517	10
Q16512	0.201840037	0.6893553	10
P10636	0.197929539	0.5896061	10
P23280	0.197823122	0.6257492	19
P46013	0.19648422	0.19648422	1
Q9NZ45	0.1961403	0.1961403	1

Table S3. KEGG signal pathway enrichment analysis

**Term**	**Count**	**%**	**PValue**	**Genes**
hsa01100:Metabolic pathways	29	29	0.000136772	PFKFB3, ALOX15, HSD17B14, AKR1B1, PIK3CB, FUT4, PIK3CG, HSD11B1, CA2, CA5A, ALOX5, HSD17B2, CA4, CA7, CD38, CA6, CA9, CA14, CA12, CBR1, ST6GAL1, NOS2, SRD5A1, PDE4D, AKR1C3, AKR1B10, TBXAS1, MGLL, PDE9A
hsa05200:Pathways in cancer	21	21	2.49435E-08	ALK, CAMK2B, NOS2, DAPK1, FLT3, MMP2, PTGER3, CXCR4, PIK3CB, F2, ESR1, IL2, PTK2, ESR2, CDC42, EDNRA, RPS6KA5, CDK6, CCNE1, PIM1, BCL2
hsa04151:PI3K-Akt signaling pathway	12	12	0.00032418	CDK6, SYK, FLT3, PDPK1, CCNE1, BCL2, KDR, PKN1, PIK3CB, PTK2, IL2, PIK3CG
hsa05022:Pathways of neurodegeneration - multiple diseases	10	10	0.026387553	CAMK2B, GRM5, CSNK2A1, NOS2, CDK5, BCL2, NOX4, MAPT, TNF, CDK5R1
hsa05205:Proteoglycans in cancer	9	9	0.00042568	CAMK2B, CDC42, PDPK1, MMP2, KDR, PIK3CB, ESR1, TNF, PTK2
hsa04020:Calcium signaling pathway	9	9	0.001769864	CAMK2B, GRM5, EDNRA, NOS2, PTGER3, KDR, CD38, CXCR4, MYLK

hsa05010:Alzheimer disease	9	9	0.021944095	GRM5, CSNK2A1, NOS2, CDK5, NOX4, MAPT, PIK3CB, TNF, CDK5R1
hsa00910:Nitrogen metabolism	8	8	5.9508E-11	CA12, CA2, CA5A, CA4, CA7, CA6, CA9, CA14
hsa04933:AGE-RAGE signaling pathway in diabetic complications	8	8	2.89478E-05	CDC42, MMP2, BCL2, PIM1, NOX4, PIK3CB, TNF, F3
hsa05170:Human immunodeficiency virus 1 infection	8	8	0.002790451	CCNB2, CCNB1, BCL2, CDK1, CXCR4, PIK3CB, TNF, PTK2
hsa05208:Chemical carcinogenesis - reactive oxygen species	8	8	0.003868797	CBR1, PDPK1, AKR1C3, CYP1B1, NOX4, AHR, PIK3CB, PTK2
hsa04210:Apoptosis	7	7	0.001306589	PARP1, PDPK1, CTSK, BCL2, GZMB, PIK3CB, TNF
hsa04218:Cellular senescence	7	7	0.002610146	CCNB2, CCNB1, CDK6, CCNE1, CDK1, PIK3CB, ZFP36L2
hsa04360:Axon guidance	7	7	0.0056828	CAMK2B, CDC42, CDK5, PDPK1, CXCR4, PIK3CB, PTK2
hsa04510:Focal adhesion	7	7	0.009048265	CDC42, PDPK1, BCL2, KDR, PIK3CB, PTK2, MYLK
hsa05203:Viral carcinogenesis	7	7	0.009470906	CDC42, CDK6, SYK, CCNE1, CDK1, PIK3CB, HDAC6
hsa05417:Lipid and	7	7	0.012051632	CAMK2B, CDC42, PDPK1, BCL2, PIK3CB, TNF, PTK2

atherosclerosis				
hsa05206:MicroRNAs in cancer	7	7	0.063641226	RPS6KA5, CDK6, CCNE1, BCL2, PIM1, CYP1B1, PIK3CB
hsa04115:p53 signaling pathway	6	6	0.000510249	CCNB2, CCNB1, CDK6, CCNE1, BCL2, CDK1
hsa05222:Small cell lung cancer	6	6	0.001357183	CDK6, NOS2, CCNE1, BCL2, PIK3CB, PTK2
hsa01522:Endocrine resistance	6	6	0.001792221	MMP2, BCL2, PIK3CB, ESR1, PTK2, ESR2
hsa05145:Toxoplasmosis	6	6	0.003074358	NOS2, PDPK1, ALOX5, BCL2, TNF, PIK3CG
hsa04722:Neurotrophin signaling pathway	6	6	0.004134185	CAMK2B, CDC42, RPS6KA5, PDPK1, BCL2, PIK3CB
hsa04611:Platelet activation	6	6	0.005084482	SYK, TBXAS1, PIK3CB, F2, PIK3CG, MYLK
hsa05135:Yersinia infection	6	6	0.007433535	CDC42, PKN1, PIK3CB, TNF, PTK2, IL2
hsa05162:Measles	6	6	0.007658378	CDK6, CSNK2A1, CCNE1, BCL2, PIK3CB, IL2
hsa04114:Oocyte meiosis	6	6	0.007658378	CAMK2B, CCNB2, CCNB1, CCNE1, CDK1, AURKA
hsa05418:Fluid shear stress and atherosclerosis	6	6	0.008361346	MMP2, BCL2, KDR, PIK3CB, TNF, PTK2

hsa04148:Efferocytosis	6	6	0.012556486	CAMK2B, AXL, ALOX5, ALOX15, NR1H3, PTK2
hsa04062:Chemokine signaling pathway	6	6	0.027939422	CDC42, GRK1, CXCR4, PIK3CB, PTK2, PIK3CG
hsa05169:Epstein-Barr virus infection	6	6	0.034938211	CDK6, SYK, CCNE1, BCL2, PIK3CB, TNF
hsa05207:Chemical carcinogenesis - receptor activation	6	6	0.042940339	BCL2, CYP1B1, AHR, PIK3CB, ESR1, ESR2
hsa05163:Human cytomegalovirus infection	6	6	0.050391996	CDK6, PTGER3, CXCR4, PIK3CB, TNF, PTK2
hsa04810:Regulation of actin cytoskeleton	6	6	0.054387465	CDC42, CXCR4, PIK3CB, F2, PTK2, MYLK
hsa05132:Salmonella infection	6	6	0.071213008	CDC42, BCL2, PKN1, PIK3CB, TNF, PIK3CG
hsa05131:Shigellosis	6	6	0.073134764	CDC42, RPS6KA5, BCL2, PIK3CB, TNF, PTK2
hsa00590:Arachidonic acid metabolism	5	5	0.002282729	CBR1, ALOX5, TBXAS1, ALOX15, AKR1C3
hsa00140:Steroid hormone	5	5	0.002418698	HSD11B1, SRD5A1, HSD17B2, AKR1C3, CYP1B1

biosynthesis				
hsa04664:Fc epsilon RI signaling pathway	5	5	0.00318287	SYK, PDPK1, ALOX5, PIK3CB, TNF
hsa04064:NF-kappa B signaling pathway	5	5	0.013855883	CSNK2A1, SYK, PARP1, BCL2, TNF
hsa05215:Prostate cancer	5	5	0.014304345	GRM5, PDPK1, CCNE1, BCL2, PIK3CB
hsa04066:HIF-1 signaling pathway	5	5	0.016190071	CAMK2B, PFKFB3, NOS2, BCL2, PIK3CB
hsa04914:Progesterone-mediated oocyte maturation	5	5	0.016684753	CCNB2, CCNB1, CDK1, PIK3CB, AURKA
hsa04670:Leukocyte transendothelial migration	5	5	0.019300268	CDC42, MMP2, CXCR4, PIK3CB, PTK2
hsa04660:T cell receptor signaling pathway	5	5	0.022757633	CDC42, PDPK1, PIK3CB, TNF, IL2
hsa04915:Estrogen signaling pathway	5	5	0.034507136	MMP2, BCL2, PIK3CB, ESR1, ESR2
hsa04072:Phospholipase D	5	5	0.042800888	GRM5, SYK, PIK3CB, F2, PIK3CG

signaling pathway				
hsa04110:Cell cycle	5	5	0.051149176	CCNB2, CCNB1, CDK6, CCNE1, CDK1
hsa04217:Necroptosis	5	5	0.052128338	CAMK2B, PARP1, ALOX15, BCL2, TNF
hsa05152:Tuberculosis	5	5	0.077448116	CAMK2B, SYK, NOS2, BCL2, TNF
hsa05168:Herpes simplex virus 1 infection	5	5	0.078668213	SYK, SRSF1, BCL2, PIK3CB, TNF
hsa00790:Folate biosynthesis	4	4	0.00186475	CBR1, AKR1B10, AKR1C3, AKR1B1
hsa00051:Fructose and mannose metabolism	4	4	0.003563397	AKR1B10, PFKFB3, HSD17B14, AKR1B1
hsa04913:Ovarian steroidogenesis	4	4	0.010805231	ALOX5, HSD17B2, AKR1C3, CYP1B1
hsa04370:VEGF signaling pathway	4	4	0.015909109	CDC42, KDR, PIK3CB, PTK2
hsa05221:Acute myeloid leukemia	4	4	0.02215828	FLT3, PIM1, PIK3CB, MPO
hsa05223:Non-small cell lung cancer	4	4	0.02665196	ALK, CDK6, PDPK1, PIK3CB

hsa01521:EGFR tyrosine kinase inhibitor resistance	4	4	0.033701097	AXL, BCL2, KDR, PIK3CB
hsa05142:Chagas disease	4	4	0.062894654	NOS2, PIK3CB, TNF, IL2
hsa05146:Amoebiasis	4	4	0.062894654	NOS2, PIK3CB, TNF, PTK2
hsa04625:C-type lectin receptor signaling pathway	4	4	0.065849844	SYK, PIK3CB, TNF, IL2
hsa04931:Insulin resistance	4	4	0.071948722	PDPK1, NR1H3, PIK3CB, TNF
hsa04725:Cholinergic synapse	4	4	0.083207178	CAMK2B, BCL2, PIK3CB, PIK3CG
hsa04650:Natural killer cell mediated cytotoxicity	4	4	0.084874417	SYK, GZMB, PIK3CB, TNF
hsa04071:Sphingolipid signaling pathway	4	4	0.093423224	PDPK1, BCL2, PIK3CB, TNF
hsa05330:Allograft rejection	3	3	0.046392653	GZMB, TNF, IL2
hsa05219:Bladder cancer	3	3	0.050763837	RPS6KA5, DAPK1, MMP2
hsa04940:Type I diabetes mellitus	3	3	0.057584956	GZMB, TNF, IL2

hsa05332:Graft-versus-host disease	3	3	0.059926051	GZMB, TNF, IL2
hsa04923:Regulation of lipolysis in adipocytes	3	3	0.095783275	PTGER3, PIK3CB, MGLL

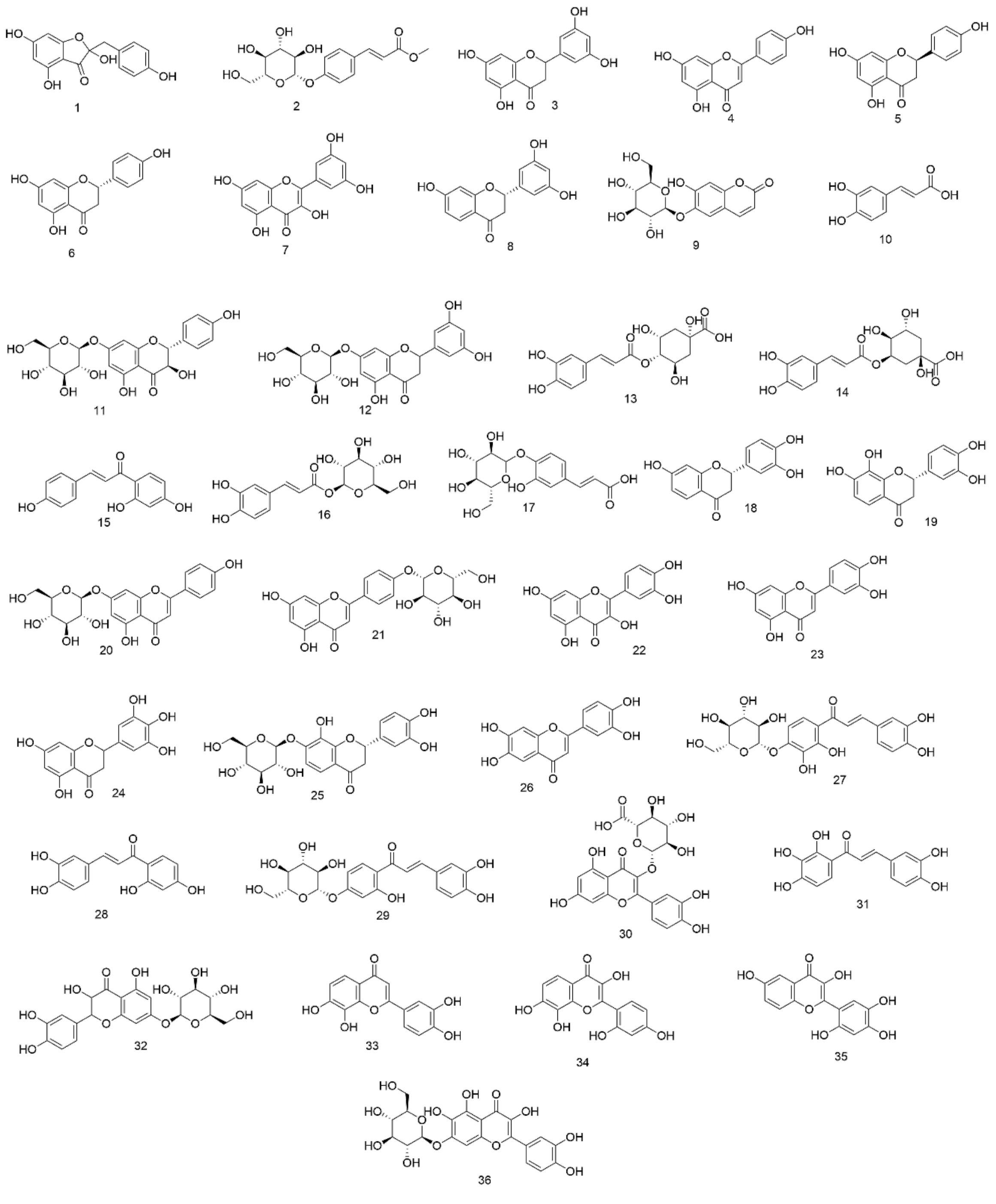

Fig S1. The structure of active compounds

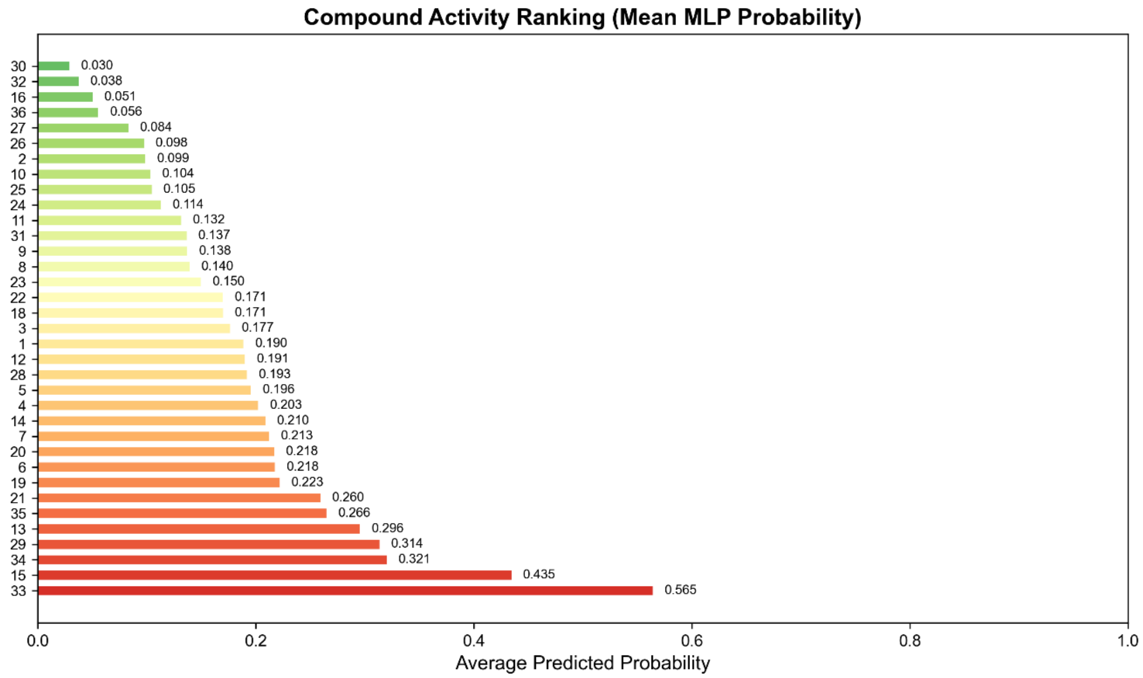

Fig S2. The complete ranking of all 36 compounds.
